# Response to ‘Impact of control selection strategies on GWAS results: a study of prostate cancer in the UK Biobank’

**DOI:** 10.1093/bib/bbag268

**Published:** 2026-05-27

**Authors:** Philip J Law, Molly Went, Richard S Houlston

**Affiliations:** Division of Genetics and Epidemiology, The Institute of Cancer Research, London, SW7 3RP, United Kingdom; Division of Genetics and Epidemiology, The Institute of Cancer Research, London, SW7 3RP, United Kingdom; Division of Genetics and Epidemiology, The Institute of Cancer Research, London, SW7 3RP, United Kingdom

Dear editor

We write to raise conceptual and methodological concerns regarding the article by Lu *et al*. (‘Impact of control selection strategies on GWAS results: a study of prostate cancer in the UK Biobank’) [1]. A central claim of the paper is that nearest-neighbour propensity score matching represents an effective, or even superior, strategy for control selection in genome-wide association studies (GWAS). We contend that this conclusion is unsupported and the core of the issue is a fundamental mismatch between the methodology employed and the aims of GWAS.

Nearest-neighbour propensity score matching is a tool developed for causal inference, where the objective is to estimate treatment effects while mitigating confounding, particularly when matching on categorical phenotypes, such as sex or socioeconomic factors. GWAS, in contrast, is an association-based framework that estimates marginal Single Nucleotide Polymorphism (SNP)–phenotype relationships using regression or mixed models that can explicitly adjust for confounding variables such as age, technical factors, and genetic principal components (PC). Within this context, matching is neither required for unbiased estimation nor theoretically motivated as a primary design strategy.

Conditioning on a variable (genotype PC) that directly influences both the exposure (genotype) and outcome (disease risk) creates a collider bias. This results in control individuals who are genetically similar to cases, thus attenuating or eliminating precisely the subtle allele-frequency differences that GWAS aims to detect. Modern GWAS tools such as REGENIE use linear mixed models, which can account for all degrees of relatedness and population structure across all the samples simultaneously. Thus, using propensity score matching on the same covariates further amplifies the overfitting effect, resulting in groups that can be so similar that the actual causal variants no longer show a statistical difference.

This risk is not merely theoretical. Recent work has shown that even the standard practice of including PCs as covariates in the association analysis can induce collider bias in certain settings [2]. More aggressive conditioning on genome-wide similarity is therefore likely to exacerbate rather than mitigate such bias.

These limitations extend to rare-variant and gene-burden analyses, where the manuscript suggests matching may be particularly advantageous. Because rare variants are strongly structured by the same fine-scale ancestry captured by genome-wide SNPs, matching implicitly conditions on the expected rare-variant spectrum and attenuates genuine signals precisely where power is already limited. Furthermore, by artificially restricting the dataset, this directly impacts the ability to identify rare variants with small effects.

In summary, nearest-neighbour matching using genotype PCs, is conceptually misaligned with the goals of GWAS. By utilizing a causal-inference tool unsuited to association testing and introducing a collider variable, the approach risks removing true biological signal, introducing bias, and sacrificing power, both for polygenic as well as rare-variant architectures. We urge the authors to clarify or correct these points to prevent misinterpretation by the field.

**Key Points**

- Propensity score matching is conceptually inappropriate for GWAS.
- Matching reduces power and using genetic principal components can induce collider bias.
- Risks from bias are empirically supported and may be worsened by aggressive matching.
